# Prolactin levels and breast cancer risk by tumor expression of prolactin-related markers

**DOI:** 10.1186/s13058-023-01618-3

**Published:** 2023-03-07

**Authors:** Cassandra A. Hathaway, Megan S. Rice, Laura C. Collins, Dilys Chen, David A. Frank, Sarah Walker, Charles V. Clevenger, Rulla M. Tamimi, Shelley S. Tworoger, Susan E. Hankinson

**Affiliations:** 1 Department of Cancer Epidemiology, Moffitt Cancer Center, 13131 Magnolia Drive, Tampa, FL 33612 USA; 2 Clinical and Translational Epidemiology Unit, Massachusetts General Hospital and Harvard Medical School, Boston, MA USA; 3 Department of Pathology, Beth Israel Deaconess Medical Center and Harvard Medical School, Boston, MA USA; 4 Royal Columbian Hospital, University of British Columbia, Vancouver, Canada; 5 Dana-Farber Cancer Institute and Harvard Medical School, Boston, MA USA; 6 Department of Hematology and Medical Oncology, Winship Cancer Institute, Emory University School of Medicine, Atlanta, GA USA; 7 Department of Pathology, Virginia Commonwealth University, Richmond, VA USA; 8 Department of Population Health Sciences, Weill Cornell Medicine, New York, NY USA; 9 Department of Biostatistics and Epidemiology, School of Public Health and Health Sciences, University of Massachusetts, Amherst, MA USA; 10 Department of Epidemiology, Harvard T.H. Chan School of Public Health, Boston, MA USA

**Keywords:** Breast cancer, Risk, Prolactin, STAT-JAK

## Abstract

**Background:**

Higher circulating prolactin has been associated with increased breast cancer risk. Prolactin binding to the prolactin receptor (PRLR) can activate the transcription factor STAT5, thus, we examined the association between plasma prolactin and breast cancer risk by tumor expression of PRLR, STAT5, and the upstream kinase JAK2.

**Methods:**

Using data from 745 cases and 2454 matched controls in the Nurses’ Health Study, we conducted polytomous logistic regression to examine the association between prolactin (> 11 ng/mL vs. ≤ 11 ng/mL) measured within 10 years of diagnosis and breast cancer risk by PRLR (nuclear [N], cytoplasmic [C]), phosphorylated STAT5 (pSTAT5; N, C), and phosphorylated JAK2 (pJAK2; C) tumor expression. Analyses were conducted separately in premenopausal (*n* = 168 cases, 765 controls) and postmenopausal women (*n* = 577 cases, 1689 controls).

**Results:**

In premenopausal women, prolactin levels > 11 ng/mL were positively associated with risk of tumors positive for pSTAT5-N (OR 2.30, 95% CI 1.02–5.22) and pSTAT5-C (OR 1.64, 95% CI 1.01–2.65), but not tumors that were negative for these markers (OR 0.98, 95% CI 0.65–1.46 and OR 0.73, 95% CI 0.43–1.25; p-heterogeneity = 0.06 and 0.02, respectively). This was stronger when tumors were positive for both pSTAT5-N and pSTAT5-C (OR 2.88, 95% CI 1.14–7.25). No association was observed for PRLR or pJAK2 (positive or negative) and breast cancer risk among premenopausal women. Among postmenopausal women, plasma prolactin levels were positively associated with breast cancer risk irrespective of PRLR, pSTAT5, or pJAK2 expression (all p-heterogeneity ≥ 0.21).

**Conclusion:**

We did not observe clear differences in the association between plasma prolactin and breast cancer risk by tumor expression of PRLR or pJAK2, although associations for premenopausal women were observed for pSTAT5 positive tumors only. While additional studies are needed, this suggests that prolactin may act on human breast tumor development through alternative pathways.

**Supplementary Information:**

The online version contains supplementary material available at 10.1186/s13058-023-01618-3.

## Background

Prolactin, a multifunctional pituitary hormone, is important in the proliferation and differentiation of normal mammary epithelium [1] and is necessary for normal lobuloalveolar development and lactation. Evidence from both laboratory [2, 3] and epidemiologic studies supports that prolactin is involved in mammary tumorigenesis [4, 5], with stronger associations for postmenopausal breast cancer that led to improvements in risk prediction models [6–8]. In a prior analysis in the Nurses’ Health Study (NHS) and NHSII we observed that plasma prolactin levels greater than 11 ng/mL measured within 10 years of diagnosis were associated with a greater risk of breast cancer among postmenopausal, but not premenopausal, women [5]. There was no association between levels of prolactin measured more than 10 years prior to diagnosis and risk.

Preclinical models suggest that prolactin may impact breast tumorigenesis through the JAK-STAT pathway. Prolactin, when bound to the prolactin receptor (PRLR), activates JAK kinases via trans-phosphorylation leading to STAT5 tyrosine phosphorylation (pSTAT5) and localization to the nucleus [9, 10]. pSTAT5 modulates expression of key target genes involved in growth, increased differentiation and survival and has been shown to have a role in resistance to antiestrogen therapy [11–16]. Further, past work has associated higher prolactin levels with increased risk of more aggressive tumors (e.g., > 2 cm, higher grade, and positive lymph nodes) [5], and pSTAT5 can be overly activated by cytokines leading to more aggressive breast cancer phenotypes, although research is limited in evaluating these associations by menopausal status [17]. The involvement of JAK-STAT signaling downstream of prolactin suggests that women with high prolactin levels may be at greater risk of developing tumors displaying activation of these markers. Such a finding would have implications to both the diagnosis and treatment of these cancers; however, no prior studies have examined this potential pathway at the population level. Therefore, in a case–control study nested in the NHS, we examined the association between circulating prolactin levels measured 10 or fewer years prior to diagnosis and risk of breast cancer by tumor expression of the PRLR, pSTAT5, and phosphorylated JAK2 (pJAK2). Given the different associations of prolactin with breast cancer risk by menopausal status, we examined these associations separately in premenopausal and postmenopausal women.

## Methods

### Study population

The NHS began in 1976 when 121,700 female registered nurses aged 30–55 years of age from 11 US states completed a baseline questionnaire. NHS participants are followed by biennially mailed questionnaires to collect information on multiple exposures, including putative breast cancer risk factors, and incident diseases, including breast cancer. In 1989–1990, 32,826 participants, ages 43–70, provided blood samples, and in 2000–2002 a subset of these women ages 53–80 (*n* = 18,743) provided a second blood sample [18]. Our study was conducted within the NHS case–control study of breast cancer nested in the subcohort of women who provided blood, as described previously [19, 20]. Briefly, breast cancer cases were matched to one or two controls on age, menopausal status at blood draw, current hormone therapy use (HT), and month, time of day, and fasting status at time of blood collection. Cases were identified through self-report on biennial questionnaires or through death records. Participants or next of kin were asked for permission to obtain their medical records, which were then reviewed to confirm disease diagnosis. For confirmed cases with a pathology report, we obtained consent from the patient or her next of kin and we requested representative paraffin-embedded tissue blocks of the breast tumor. The primary tumor block for each case diagnosed after blood draw but before 2006 was selected by a breast pathologist, who circled the tumor on the slide; eligible blocks from cases who had a pre-diagnosis blood sample were sent to the Dana-Farber/Harvard Cancer Center Specialized Histopathology Services Core for tissue microarray (TMA) construction. TMAs included three 0.6 mm cores per case.

### Ethical statement

The NHS study protocol was approved by the institutional review boards of the Brigham and Women’s Hospital and Harvard T.H. Chan School of Public Health, and those of participating registries as required. Participants provided implied consent by returning the self-administered mailed study questionnaires and blood sample; individuals in this study signed a medical record and tissue release form. This study was approved by the Mass General Brigham IRB (Protocol Number: 1999P010982).

### Assessment of prolactin levels

As described previously, prolactin was assayed by microparticle enzyme immunoassay in 12 batches at the Reproductive Endocrinology Unit Laboratory at the Massachusetts General Hospital using the AxSYM immunoassay system (Abbott Diagnostics, Chicago, IL) or by Christopher Longcope, MD (University of Massachusetts Medical Center, Worcester, MA), using the IMx System (Abbott Laboratory, Abbott Park, IL) [5]. The correlation between the two laboratories was 0.91 and across different batches within the same dataset was greater than 0.95 [5]. The coefficient of variation from blinded replicate samples was < 12% across all batches. However, mean prolactin concentrations of quality control samples varied somewhat by batch. Therefore, we adjusted prolactin levels for assay batch using the methods outlined by Rosner et al. [21], as in prior analyses [5, 22]. Briefly, batch correction was done using an average batch recalibration by performing a linear regression with prolactin levels as the dependent variable along with batch indicators and variables associated with prolactin levels (e.g., menopausal status) as the independent variables. We then used the intercept and β coefficient to rescale the original prolactin values [22].

### Assessment of breast cancer risk factors

Participants were queried on age at menarche and height on the 1976 baseline questionnaire. Current weight and personal history of benign breast disease (BBD) were assessed at baseline and on all biennial questionnaires. Hormone therapy (HT) use and fasting status at the time of blood collection were assessed using a questionnaire administered to participants at blood collection. Body mass index (BMI) was calculated as weight (kg)/height (m)^2^. For covariates with repeated measures throughout follow-up, we used data from the questionnaire cycle closest in time prior to blood collection.

### Assessment of tumor expression

Freshly cut sections of the TMA blocks were assayed by immunohistochemistry (IHC) at the laboratory of Charles Clevenger at Northwestern University for the following markers: (1) prolactin receptor (PRLR) (clone: Rb/NGS, Manufacturer: SCBT, Dilution: 1/250, Catalog Number: 35-92000, Invitrogen), (2) tyrosine phosphorylated STAT5 (pSTAT5) (clone: Rb/NGS, Manufacturer: Abcam, Dilution: 1/100, Catalog Number: 9359S, Cell Signaling Technology) and (3) pJAK2 (clone: Rb/BGS, Manufacturer: Abcam, Dilution: 1/40, Catalog Number: 3776S, Cell Signaling Technology). Study pathologists (LC, DC), blinded to participant identity and characteristics, scored each TMA core, and determined tumor area morphologically as tumor cells are readily differentiated from cells in the tumor microenvironment, either based on visual differential or using the H&E stained slide. For PRLR and pSTAT5, cores were scored from 0 to 3 (none, low, medium, high) for cytoplasmic and nuclear staining intensity separately. A case was considered positive if one or more cores were scored 2 or 3, except for cytoplasmic pSTAT5, which was scored positive if any core was scored 1 or higher as few cores were scored 2 or 3. pJAK2 was scored from 0 to 2 (negative, low positive, high positive) in the cytoplasm. The intraclass correlations across cores ranged from 0.78 to 0.88. A case was considered positive for pJAK2 if any core was scored 1 or 2. For 3.0–12.6% of cases, depending on the stain, intensity was unable to be assessed (e.g., limited tumor tissue was observable by the pathologist on the cores) and are excluded from the analysis.

### Statistical analysis

Polytomous unconditional logistic regression was used to estimate odds ratios (OR) and 95% confidence intervals (CI) as well as to test for heterogeneity by tumor expression (positive versus negative as described above). Based on our prior research [5], we dichotomized circulating prolactin levels and compared levels greater than 11 ng/mL to levels 11 ng/mL or less. Using covariate status at the time of relevant blood collection, we adjusted for age (continuous), age at menarche (continuous), BMI (continuous), history of BBD (never, confirmed by biopsy, not confirmed or biopsy status unknown), hormone therapy [HT] use (postmenopausal only: no, yes), and blood draw characteristics (fasting status and month of blood collection). Further adjusting for breastfeeding (continuous) and parity (nulliparous, 1 child, 2 children, 3 children, 4 or more children) did not substantially change the results. All analyses were conducted separately in premenopausal and postmenopausal women given the different associations previously observed in these populations [5]. *P* values were 2-sided and considered statistically significant if less than 0.05. All analyses were conducted using SAS, version 9.4 (SAS Institute Inc., Cary, North Carolina).

## Results

In total, 745 cases and 2454 controls were included (Table 1); 168 cases and 765 controls were premenopausal at the time of blood collection and 577 cases and 1689 controls were postmenopausal. Cases were more likely to have a history of BBD and to have a family history of breast cancer, though they were similar to controls on other factors (e.g., age at menarche and BMI). Overall, participant and tumor characteristics were similar by prolactin-related tumor expression (Additional file 1: Table S1, Table S2).

**Table 1:** Selected characteristics of the study population by menopausal status and case status (Nurses’ Health Study, 1989–2006)

Population characteristics	Premenopausal (168 cases/765 controls)		Postmenopausal (577 cases/1689 controls)	
	Mean (SD)		Mean (SD)	
Age at blood collection*	49.5 (3.8)	49.6 (3.5)	62.7 (6.5)	62.7 (6.3)
Age at menarche	12.3 (1.4)	12.4 (1.3)	12.5 (1.4)	12.6 (1.4)
BMI at blood collection	24.7 (4.0)	25.5 (4.7)	26.0 (4.7)	25.8 (4.5)
Prolactin (ng/mL)	15.1 (9.2)	14.3 (8.8)	12.0 (7.7)	11.4 (7.7)
Breastfeeding (months)	9.1 (12.0)	7.6 (10.9)	7.1 (10.9)	5.7 (9.3)
Time from blood collection to diagnosis (years)	6.0 (3.0)		4.7 (3.1)	

	*N* (%)		*N* (%)	
Fasting at blood collection*	106 (63.1)	481 (62.9)	411 (71.2)	1205 (71.3)
Family history of breast cancer	18 (10.7)	63 (8.2)	99 (17.2)	224 (13.3)
Parity				
Nulliparous	12 (7.1)	45 (5.9)	37 (6.4)	103 (6.1)
1 child	12 (7.1)	50 (6.5)	37 (6.4)	105 (6.2)
2 children	52 (31.0)	267 (34.9)	143 (24.8)	372 (22.0)
3 children	57 (33.9)	243 (31.8)	168 (29.1)	466 (27.6)
4 or more children	35 (20.8)	160 (20.9)	192 (33.3)	643 (38.1)
History of BBD				
Biopsy-confirmed	37 (22.0)	124 (16.2)	124 (21.5)	276 (16.3)
Unconfirmed or confirmation unknown	64 (38.1)	251 (32.8)	188 (32.6)	485 (28.7)
HT use at blood collection*	–	–	344 (59.6)	721 (42.7)
Invasive status				
Invasive	140 (83.3)		494 (85.6)	
DCIS	28 (16.7)		83 (14.4)	
ER status				
Negative	30 (17.9)		103 (17.9)	
Positive	137 (81.5)		462 (80.1)	
Unknown	1 (0.6)		12 (2.1)	
PR status				
Negative	44 (26.2)		148 (25.6)	
Positive	124 (73.8)		422 (73.1)	
Unknown	–		7 (1.2)	
HER-2 status				
Negative	110 (65.5)		433 (75.0)	
Positive	54 (32.1)		127 (22.0)	
Unknown	4 (2.4)		17 (2.9)	
Tumor marker (score)				
PRLR nuclear				
Negative (0/1)	134 (79.8)		406 (70.4)	
Positive (2/3)	29 (17.3)		139 (24.1)	
Unknown	5 (3.0)		32 (5.5)	
PRLR cytoplasmic				
Negative (0/1)	128 (76.2)		416 (72.1)	
Positive (2/3)	35 (20.8)		129 (22.4)	
Unknown	5 (3.0)		32 (5.5)	
pSTAT5 nuclear				
Negative (0/1)	117 (69.6)		394 (68.3)	
Positive (2/3)	35 (20.8)		125 (21.7)	
Unknown	16 (9.5)		58 (10.1)	
pSTAT5 cytoplasmic				
Negative (0)	61 (36.3)		213 (36.9)	
Positive (1/2)	91 (54.2)		306 (53.0)	
Unknown	16 (9.5)		58 (10.1)	
pJAK2				
Negative (0)	20 (11.9)		95 (16.5)	
Positive (1/2)	127 (75.6)		409 (70.9)	
Unknown	21 (12.5)		73 (12.7)	

*BMI* body mass index, *BBD* benign breast disease, *DCIS* ductal carcinoma in situ, *HT* hormone therapy

*Matching factor; cases who were not using HT at blood collection were matched to two controls and cases using HT at blood collection were matched to one control

Among all premenopausal women, a nonsignificant positive association was observed comparing prolactin levels of > 11 ng/mL vs. ≤ 11 ng/mL. When we evaluated associations by tumor receptor status, we observed a significant increase in risk for prolactin levels > 11 vs. ≤ 11 ng/mL for pSTAT5 nuclear positive (OR 2.30, 95% CI 1.02, 5.22, p-heterogeneity: 0.06) but not pSTAT5 nuclear negative tumors (OR 0.98, 95% CI 0.65, 1.46). Similar results were observed for cytoplasmic pSTAT5 (OR stain positive: 1.64, 95% CI 1.01, 2.65; OR stain negative: 0.73, 95% CI 0.43, 1.25; p-heterogeneity: 0.02) tumors (Table 2; Additional file 1: Figure S1A–1C). Tumors which were positive for both pSTAT5 nuclear and cytoplasmic had an increase in risk for prolactin levels > 11 ng/mL (comparable OR 2.88, 95% CI 1.14, 7.25; p-heterogeneity: 0.03) which was not observed if only pSTAT5 nuclear or cytoplasmic was positive (OR 1.24, 95% CI 0.72, 2.12), or if both were negative (OR 0.73, 95% CI 0.42, 1.28). There was no association between plasma prolactin levels and breast cancer risk by tumor expression of PRLR (nuclear or cytoplasmic) or pJAK2.

**Table 2:** Odds ratios (ORs) and 95% confidence intervals (CIs) for breast cancer comparing plasma prolactin ≥ 11 versus < 11 ng/mL by tumor expression of prolactin-related markers among premenopausal women

Tumor status	*N* (Cases)	Prolactin levels (ng/mL)		p-heterogeneity
		≤ 11	> 11	
All breast cancer	168	1 (Ref)	1.22 (0.85, 1.73)	
Prolactin receptor				
Nuclear^a^				
Negative	134	1 (Ref)	1.24 (0.84, 1.83)	0.92
Positive	29	1 (Ref)	1.19 (0.54, 2.60)	
Cytoplasmic^a^				
Negative	128	1 (Ref)	1.37 (0.92, 2.05)	0.22
Positive	35	1 (Ref)	0.84 (0.42, 1.69)	
pSTAT5				
Nuclear^a^				
Negative	117	1 (Ref)	0.98 (0.65, 1.46)	0.06
Positive	35	1 (Ref)	**2.30 (1.02, 5.22)**	
Cytoplasmic^b^				
Negative	61	1 (Ref)	0.73 (0.43, 1.25)	**0.02**
Positive	91	1 (Ref)	**1.64 (1.01, 2.65)**	
Combined nuclear/cytoplasmic				
Both negative	56	1 (Ref)	0.73 (0.42, 1.28)	**0.03**
Both positive	30	1 (Ref)	**2.88 (1.14, 7.25)**	
One negative/one positive	66	1 (Ref)	1.24 (0.72, 2.12)	
pJAK2^b^				
Negative	20	1 (Ref)	1.72 (0.64, 4.65)	0.49
Positive	127	1 (Ref)	1.20 (0.80, 1.78)	

Bolded values are statistically significant (*p* < 0.05)

Adjusted for age (continuous), age at menarche (continuous), BMI (continuous), benign breast disease (never, confirmed, unconfirmed), and blood draw characteristics (fasting status and month of blood collection)

^a^A case was considered positive if one or more cores were scored 2 or 3

^b^Cytoplasmic pSTAT5 and pJAK2 was scored positive if any core was scored 1 or higher

In postmenopausal women, plasma prolactin levels > 11 vs. ≤ 11 ng/mL were significantly associated with a higher risk of breast cancer overall, with no significant differences by PRLR or pSTAT5 tumor expression status (Table 3; Additional file 1: Figure S1A–1C). While plasma prolactin levels were suggestively more strongly associated with pSTAT5 nuclear positive tumors (OR 1.69, 95% CI 1.16, 2.46) than pSTAT5 nuclear negative tumors (OR 1.29, 95% CI 1.03, 1.63), this difference was not statistically significant (p-heterogeneity: 0.21). For tumors with positive pJAK2 expression, high prolactin levels were significantly associated with risk of breast cancer with positive pJAK2 expression (comparable OR 1.38, 95% CI 1.10, 1.73) and suggestively associated for pJAK2 negative tumors (comparable OR 1.52, 95% CI 0.99, 2.33, p-heterogeneity = 0.68).

**Table 3:** Odds ratios (ORs) and 95% confidence intervals (CIs) for breast cancer comparing plasma prolactin ≥ 11 versus < 11 ng/mL by tumor expression of prolactin-related markers among postmenopausal women

Tumor status	*N* (Cases)	Prolactin levels (ng/mL)		p-heterogeneity
		≤ 11	> 11	
All breast cancer	577	1(Ref)	**1.37 (1.12, 1.67)**	
Prolactin receptor				
Nuclear^a^				
Negative	406	1 (Ref)	**1.32 (1.05, 1.66)**	0.65
Positive	139	1 (Ref)	**1.45 (1.01, 2.07)**	
Cytoplasmic^a^				
Negative	416	1 (Ref)	**1.32 (1.05, 1.65)**	0.60
Positive	129	1 (Ref)	**1.47 (1.01, 2.14)**	
pSTAT5				
Nuclear^a^				
Negative	394	1 (Ref)	**1.29 (1.03, 1.63)**	0.21
Positive	125	1 (Ref)	**1.69 (1.16, 2.46)**	
Cytoplasmic^b^				
Negative	213	1 (Ref)	**1.43 (1.07, 1.92)**	0.73
Positive	306	1 (Ref)	**1.34 (1.04, 1.73)**	
Combined nuclear/cytoplasmic				
Both negative	189	1 (Ref)	**1.45 (1.06, 1.97)**	0.53
Both positive	101	1 (Ref)	**1.78 (1.18, 2.68)**	
One negative/one positive	229	1 (Ref)	**1.35 (1.02, 1.80)**	
pJAK2^b^				
Negative	95	1 (Ref)	1.52 (0.99, 2.33)	0.68
Positive	409	1 (Ref)	**1.38 (1.10, 1.73)**	

Bolded values are statistically significant (*p* < 0.05)

Adjusted for age (continuous), age at menarche (continuous), BMI (continuous), benign breast disease (never, confirmed, unconfirmed), HT status (no, yes), and blood draw characteristics (fasting status and month of blood collection)

^a^A case was considered positive if one or more cores were scored 2 or 3

^b^Cytoplasmic pSTAT5 and pJAK2 was scored positive if any core was scored 1 or higher

When we examined risk of breast cancer by joint tumor expression in postmenopausal women, we observed that plasma prolactin levels were more strongly associated with a risk of tumors that were positive for both PRLR nuclear and cytoplasmic expression (OR > 11 vs. ≤ 11 ng/mL: 5.90, 95% CI 1.90, 18.30, p-heterogeneity: 0.05) compared to the other three subtypes (Additional file 1: Table S3), although only 21 cases were positive for both PRLR nuclear and cytoplasmic expression. No significant heterogeneity of the prolactin/breast cancer association was observed for any of the other combinations of two tumor markers (p-heterogeneity: 0.07 to 0.88). The results were similar when restricting to only invasive tumors (Additional file 1: Table S4 and S5) and among postmenopausal women with ER+/PR+ tumors (Additional file 1: Table S6). The sample size among premenopausal women was too small to assess joint associations in that subset. We evaluated tumor characteristics by PRLR, pSTAT5, and JAK2 expression among premenopausal (Additional file 1: Table S7) and postmenopausal (Additional file 1: Table S8) women. Overall, premenopausal women had slightly more tumor differences (e.g., tumor grade and tumor size) by PRLR, pSTAT5, and pJAK2 expression than postmenopausal women; however, the sample size was limited.

## Discussion

We examined the potential etiologic role of prolactin in the development of breast cancer within a large case–control study nested in a prospective cohort of women with pre-diagnosis blood and breast tumor tissue. Overall, we did not observe clear differences in the association of circulating prolactin levels and risk of breast cancer by expression of PRLR or downstream markers of prolactin receptor activation, pJAK2 and pSTAT5. However, among premenopausal women, those with modestly high versus lower prolactin levels (although still within the normal range) had an increased risk of tumors with either nuclear or cytoplasmic pSTAT5 expression (or both), but not of tumors with limited pSTAT5 staining. Consistent with our prior work, which included the women in this study, the overall associations were stronger in postmenopausal compared to premenopausal women [5].

Despite an overall lack of association between circulating prolactin levels and risk of breast cancer in premenopausal women [5], we observed that prolactin was positively related to a higher risk of pSTAT5 positive breast cancer in this population. This suggests that prolactin may act through the JAK-STAT pathway with respect to premenopausal breast carcinogenesis, although the number of cases in the analysis was modest. STAT5 activation, a downstream effect of prolactin binding to the PRLR, is involved in regulation of mammary gland development, mediating the effects of estrogen and progesterone on this process [23–25]. Constitutive STAT5 activation is oncogenic, promoting tumor initiation through overexpression of transforming growth factor-α (TGFα) and increased cellular proliferation [23, 24], although among women with estrogen receptor (ER) positive breast cancer, the presence of pSTAT5 was associated with better response to hormone therapy and improved survival [16]. A recent study has shown ERα-regulated genes and prolactin regulated genes largely overlap, with stronger overlap for prolactin regulated genes with promoters or enhancers of STAT5a, suggesting an increase in estrogen may play a mediating role in the associations we observed in premenopausal women [26]. Further work is needed to understand the direct effects of STAT5 on ER status and future breast cancer development. Although pSTAT5 can be activated through the PRLR pathway, we did not see the same increased risk of a PRLR positive breast cancer with elevated prolactin. This may be due to the isoform used to measure PRLR, potentially indicating another isoform of PRLR is important. Alternatively, this could indicate pSTAT5 is being activated in premenopausal breast cancer through a mechanism other than PRLR [27], which should be explored in future research.

Conversely, among postmenopausal women, prolactin was similarly associated with breast cancer risk regardless of pSTAT5 expression or expression of PRLR or pJAK2, suggesting that this pathway may not be the sole or even primary mechanism of action in this population. One potential alternative mechanism may be through the JAK2-nuclear factor 1-C2 (NF1-C2) pathway [28–30]. PRLR activates JAK2 that in turn can activate NF1-C2 in the nucleus, independent from STAT5 activation [28–30]. NF1-C2 has been shown to activate the TP53 tumor suppressor gene and prolactin may mediate this relationship [29]. In addition to NF1-C2, the prolactin can activate other pathways such as SRC family kinases, AKT, and MAP kinases [31]. The multiple pathways downstream of JAK2 and the fact that other growth factors and hormones can activate this pathway may explain why we did not observe a specific association by pJAK2 tumor expression. Overall, more research is needed to evaluate NF1-C2 and other pathways activated by prolactin in the postmenopausal, low hormone context.

In addition to STAT5, the other STAT family member activating commonly in breast cancer is STAT3 [32]. There is evidence from both human cancers and model systems that the activation of STAT5 is associated with less aggressiveness and increased sensitivity to chemotherapy of breast cancers that also display activation of STAT3 [33], and that this may be mediated by repression by STAT5 of genes that are otherwise upregulated by STAT3 [34]. Thus, understanding the activation state of STAT3 as well as STAT5 might provide insight into the current findings. Furthermore, as therapies directed toward these transcription factors are being developed, an understanding of the association between hormonal milieu and the risk and biology of these cancers becomes increasingly important [35].

This study has several strengths, including a large sample size, pre-diagnosis measures of prolactin, and associated tumor tissue. Further, we were able to evaluate nuclear and cytoplasmic tumor cell compartments for staining of PRLR and pSTAT5 to evaluate differences by cellular location of marker expression. However, we were unable to evaluate pSTAT5a and pSTAT5b expression separately. Both are stimulated by prolactin and play a similar role in mammary gland development; however they differ in their downstream effects in the immune response, such that pSTAT5a is important for T-helper cells, and pSTAT5b plays a role in proliferation of natural killer cells [36, 37]. Therefore, future studies should assess the variation in breast cancer risk by pSTAT5a and pSTAT5b, particularly in premenopausal women. Additionally, we evaluated tyrosine phosphorylated STAT5, however unphosphorylated STAT5 as well as serine-phosphorylated STAT5 may have varying associations with breast cancer risk and should be considered in future studies [38, 39]. Similarly, our assessment of PRLR did not distinguish between different isoforms of this receptor, which have been shown to have differential effects on downstream STAT5 signaling [40], suggesting that future studies should separately evaluate the most oncogenic isoforms. Tumor expression of PRLR, STAT5 and JAK2 was read manually by pathologist that may have led to measurement error; therefore, future studies should consider using image analysis to reduce subjectivity. Further, larger studies are needed to assess joint associations of biologically relevant tumor markers including of other prolactin-related pathways. We observed some differences in tumor characteristics (i.e., ductal/lobular, lymph node involvement, tumor grade, and tumor size) by PRLR, pSTAT5 and pJAK2 expression and menopausal status. The role of PRLR and pSTAT5 in tumors after development remains unclear, and more work is needed to understand the biological importance of these markers and their clinical relevance [31].

## Conclusion

Overall, we found that pSTAT5-positive breast cancer risk was increased for premenopausal women with high prolactin levels, suggesting that prolactin may be causally related to breast cancer risk in a subset of women through the traditional prolactin-related JAK-STAT pathway. However, no differences in risk were observed by expression of pSTAT5, PRLR, or pJAK2 in postmenopausal women, for whom prolactin was consistently positively associated with risk. This suggests that prolactin may be related to another factor that alters breast cancer risk or that it acts through other biologic pathways, such as NF1-C2, which have yet to be fully elucidated. Given the large variation in the hormonal milieu between premenopausal and postmenopausal women, better understanding of the differential actions of prolactin under low and high estrogen and progesterone conditions may provide further insight into these relationships.

## Supplementary Information

**Additional file 1.** Supplemental tables and figure.

## Data Availability

The datasets used and analyzed during the current study are available from the corresponding author on reasonable request.

## Abbreviations

BMI: Body mass index
BBD: Benign breast disease
C: Cytoplasmic
CI: Confidence intervals
ER: Estrogen receptor
HT: Hormone therapy
IHC: Immunohistochemistry
N: Nuclear
NF1-C2: JAK2-nuclear factor 1-C2
NHS: Nurses’ Health Study
OR: Odds ratios
pJAK2: Phosphorylated JAK2
PRLR: Prolactin receptor
pSTAT5: STAT5 tyrosine phosphorylation
TGFα: Transforming growth factor-α
TMA: Tissue microarray
