# Supplementary material for: Prolactin levels and breast cancer risk by tumor expression of prolactin-related markers
Source: Breast Cancer Res. 2023 Mar 7;25:24. doi: 10.1186/s13058-023-01618-3 (PMC9990334; doi:10.1186/s13058-023-01618-3)
Supplement: Supplementary file 1 — Additional file 1. Supplemental tables and figure. [file 13058_2023_1618_MOESM1_ESM.docx]

**Supplementary Table 1.** Participant and tumor characteristics by case and tumor expression status among premenopausal women

|  |  | **Tumor Characteristics** | | | | | | | | | |
| --- | --- | --- | --- | --- | --- | --- | --- | --- | --- | --- | --- |
| **Participant Characteristics** | **Controls** | **Prolactin receptor Nuclear** | | **Prolactin receptor Cytoplasmic** | | **pSTAT5**  **Nuclear** | | **pSTAT5**  **Cytoplasmic** | | **pJAK2** | |
|  | **N=765** | **Positive (2/3)**  **N=29** | **Negative (0/1)**  **N=134** | **Positive (2/3)**  **N=35** | **Negative (0/1)**  **N=128** | **Positive (2/3)**  **N=35** | **Negative (0/1)**  **N=117** | **Positive (1/2)**  **N=91** | **Negative (0)**  **N=61** | **Positive (1/2)**  **N=127** | **Negative (0)**  **N=20** |
|  | **Mean (SD)** | | | | | | | | | | |
| Age at blood collection | 49.6(3.5) | 49.8(4.1) | 49.3(3.8) | 49.6(3.7) | 49.3(3.9) | 50.1(4.0) | 49.3(3.8) | 49.3(3.9) | 49.7(3.8) | 49.8(3.8) | 48.7(4.0) |
| Age at menarche | 12.4(1.3) | 12.3(1.3) | 12.3(1.4) | 12.1(1.5) | 12.3(1.4) | 12.2(1.1) | 12.3(1.5) | 12.3(1.4) | 12.3(1.5) | 12.3(1.4) | 12.2(1.5) |
| BMI at blood collection | 25.5(4.7) | 24(3.4) | 24.7(4.0) | 24.6(4.2) | 24.6(3.9) | 24.5(3.1) | 24.6(4.3) | 24.3(3.7) | 25(4.5) | 25(4.2) | 23.4(2.4) |
| Prolactin (ng/mL) | 14.3(8.8) | 15.7(13.7) | 15(8.1) | 12.4(5.1) | 15.9(10.1) | 16.8(12) | 14.3(8.5) | 15.8(9.5) | 13.5(9.3) | 15(9.2) | 18(12.1) |
|  | **N (%)** | | | | | | | | | | |
| Fasting at blood collection | 481(62.9) | 14(48.3) | 89(66.4) | 23(65.7) | 80(62.5) | 23(65.7) | 71(60.7) | 58(63.7) | 36(59) | 84(66.1) | 10(50.0) |
| Family history of breast cancer | 63(8.2) | 4(13.8) | 13(9.7) | 2(5.7) | 15(11.7) | 6(17.1) | 10(8.5) | 7(7.7) | 9(14.8) | 14(11.0) | -- |
| History of BBD |  |  |  |  |  |  |  |  |  |  |  |
| Biopsy-confirmed | 124(16.2) | 9(31.0) | 28(20.9) | 8(22.9) | 29(22.7) | 7(20.0) | 29(24.8) | 22(24.2) | 14(23) | 31(24.4) | 5(25.0) |
| Unconfirmed or confirmation unknown | 251(32.8) | 7(24.1) | 53(39.6) | 13(37.1) | 47(36.7) | 12(34.3) | 44(37.6) | 32(35.2) | 24(39.3) | 50(39.4) | 4(20.0) |
| **Tumor characteristics** |  |  |  |  |  |  |  |  |  |  |  |
| Invasiveness |  |  |  |  |  |  |  |  |  |  |  |
| In situ |  | 4(13.8) | 24(17.9) | 7(20.0) | 21(16.4) | 6(17.1) | 20(17.1) | 16(17.6) | 10(16.4) | 23(18.1) | 2(10.0) |
| Invasive |  | 25(86.2) | 108(80.6) | 28(80.0) | 105(82.0) | 28(80.0) | 96(82.1) | 73(80.2) | 51(83.6) | 103(81.1) | 17(85.0) |
| Unknown |  | -- | 2(1.5) | -- | 2(1.6) | 1(2.9) | 1(0.9) | 2(2.2) | -- | 1(0.8) | 1(5.0) |
| Differentiation |  |  |  |  |  |  |  |  |  |  |  |
| Well or moderately differentiated |  | 13(44.8) | 47(35.1) | 10(28.6) | 50(39.1) | 14(40.0) | 42(35.9) | 34(37.4) | 22(36.1) | 47(37.0) | 6(30.0) |
| Poorly differentiated |  | 3(10.3) | 34(25.4) | 15(42.9) | 22(17.2) | 8(22.9) | 28(23.9) | 18(19.8) | 18(29.5) | 27(21.3) | 8(40.0) |
| Unknown |  | 13(44.8) | 53(39.6) | 10(28.6) | 56(43.8) | 13(37.1) | 47(40.2) | 39(42.9) | 21(34.4) | 53(41.7) | 6(30.0) |
| Lymph node status |  |  |  |  |  |  |  |  |  |  |  |
| No lymph nodes |  | 16(55.2) | 75(56) | 19(54.3) | 72(56.3) | 23(65.7) | 60(51.3) | 50(54.9) | 33(54.1) | 74(58.3) | 9(45.0) |
| At least 1 lymph node involved |  | 9(31) | 33(24.6) | 9(25.7) | 33(25.8) | 5(14.3) | 36(30.8) | 23(25.3) | 18(29.5) | 29(22.8) | 8(40.0) |
| Unknown |  | 4(13.8) | 26(19.4) | 7(20.0) | 23(18) | 7(20.0) | 21(17.9) | 18(19.8) | 10(16.4) | 24(18.9) | 3(15.0) |
| Tumor size |  |  |  |  |  |  |  |  |  |  |  |
| <2 cm |  | 17(58.6) | 78(58.2) | 24(68.6) | 71(55.5) | 22(62.9) | 69(59) | 50(54.9) | 41(67.2) | 77(60.6) | 11(55.0) |
| ≥2cm |  | 6(20.7) | 28(20.9) | 4(11.4) | 30(23.4) | 5(14.3) | 25(21.4) | 21(23.1) | 9(14.8) | 23(18.1) | 5(25.0) |
| Unknown |  | 6(20.7) | 28(20.9) | 7(20.0) | 27(21.1) | 8(22.9) | 23(19.7) | 20(22.0) | 11(18.0) | 27(21.3) | 4(20.0) |
| Ductal vs lobular |  |  |  |  |  |  |  |  |  |  |  |
| Ductal |  | 18(62.1) | 92(68.7) | 26(74.3) | 84(65.6) | 26(74.3) | 77(65.8) | 60(65.9) | 43(70.5) | 86(67.7) | 13(65) |
| Lobular |  | 7(24.1) | 12(9) | 1(2.9) | 18(14.1) | 1(2.9) | 15(12.8) | 12(13.2) | 4(6.6) | 15(11.8) | 3(15) |
| Unknown |  | 4(13.8) | 30(22.4) | 8(22.9) | 26(20.3) | 8(22.9) | 25(21.4) | 19(20.9) | 14(23) | 26(20.5) | 4(20) |
| Estrogen receptor |  |  |  |  |  |  |  |  |  |  |  |
| Negative |  | 2(6.9) | 27(20.1) | 11(31.4) | 18(14.1) | 5(14.3) | 24(20.5) | 13(14.3) | 16(26.2) | 17(13.4) | 7(35.0) |
| Positive |  | 27(93.1) | 107(79.9) | 24(68.6) | 110(85.9) | 29(82.9) | 93(79.5) | 77(84.6) | 45(73.8) | 110(86.6) | 13(65.0) |
| Unknown |  | -- | -- | -- | -- | 1(2.9) | -- | 1(1.1) | -- |  |  |
| Progesterone receptor |  |  |  |  |  |  |  |  |  |  |  |
| Negative |  | 3(10.3) | 40(29.9) | 13(37.1) | 30(23.4) | 6(17.1) | 37(31.6) | 24(26.4) | 19(31.1) | 29(22.8) | 9(45.0) |
| Positive |  | 26(89.7) | 94(70.1) | 22(62.9) | 98(76.6) | 29(82.9) | 80(68.4) | 67(73.6) | 42(68.9) | 98(77.2) | 11(55.0) |
| Unknown |  | -- | -- | -- | -- |  |  |  |  |  |  |
| HER-2 |  |  |  |  |  |  |  |  |  |  |  |
| Negative |  | 21(72.4) | 88(65.7) | 20(57.1) | 89(69.5) | 22(62.9) | 79(67.5) | 53(58.2) | 48(78.7) | 90(70.9) | 6(30.0) |
| Positive |  | 8(27.6) | 45(33.6) | 15(42.9) | 38(29.7) | 12(34.3) | 38(32.5) | 38(41.8) | 12(19.7) | 36(28.3) | 13(65.0) |
| Unknown |  | -- | 1(0.7) | -- | 1(0.8) | 1(2.9) | -- | -- | 1(1.6) | 1(0.8) | 1(5.0) |
| Prolactin Receptor Nuclear |  |  |  |  |  |  |  |  |  |  |  |
| Negative (0/1) |  | -- | -- | 33(94.3) | 101(78.9) | 23(65.7) | 97(82.9) | 69(75.8) | 51(83.6) | 102(80.3) | 16(80.0) |
| Positive (2/3) |  | -- | -- | 2(5.7) | 27(21.1) | 10(28.6) | 19(16.2) | 20(22.0) | 9(14.8) | 24(18.9) | 2(10.0) |
| Unknown |  | -- | -- | -- | -- | 2(5.7) | 1(0.9) | 2(2.2) | 1(1.6) | 1(0.8) | 2(10.0) |
| Prolactin Receptor Cytoplasmic |  |  |  |  |  |  |  |  |  |  |  |
| Negative (0/1) |  | 27(93.1) | 101(75.4) | -- | -- | 21(60) | 94(80.3) | 65(71.4) | 50(82.0) | 95(74.8) | 16(80.0) |
| Positive (2/3) |  | 2(6.9) | 33(24.6) | -- | -- | 12(34.3) | 22(18.8) | 24(26.4) | 10(16.4) | 31(24.4) | 2(10.0) |
| Unknown |  | -- | -- | -- | -- | 2(5.7) | 1(0.9) | 2(2.2) | 1(1.6) | 1(0.8) | 2(10.0) |
| pSTAT5 Nuclear |  |  |  |  |  |  |  |  |  |  |  |
| Negative (0/1) |  | 19(65.5) | 97(72.4) | 22(62.9) | 94(73.4) | -- | -- | 61(67.0) | 56(91.8) | 90(70.9) | 19(95.0) |
| Positive (2/3) |  | 10(34.5) | 23(17.2) | 12(34.3) | 21(16.4) | -- | -- | 30(33.0) | 5(8.2) | 30(23.6) | -- |
| Unknown |  | 0(0) | 14(10.4) | 1(2.9) | 13(10.2) | -- | -- | -- | -- | 7(5.5) | 1(5.0) |
| pSTAT5 Cytoplasmic |  |  |  |  |  |  |  |  |  |  |  |
| Negative (0) |  | 9(31) | 51(38.1) | 10(28.6) | 50(39.1) | 5(14.3) | 56(47.9) | -- | -- | 45(35.4) | 10(50.0) |
| Positive (1/2) |  | 20(69) | 69(51.5) | 24(68.6) | 65(50.8) | 30(85.7) | 61(52.1) | -- | -- | 75(59.1) | 9(45.0) |
| Unknown |  | 0(0) | 14(10.4) | 1(2.9) | 13(10.2) | -- | -- | -- | -- | 7(5.5) | 1(5.0) |
| pJAK2 |  |  |  |  |  |  |  |  |  |  |  |
| Negative (0) |  | 2(6.9) | 16(11.9) | 2(5.7) | 16(12.5) | -- | 19(16.2) | 9(9.9) | 10(16.4) | -- | -- |
| Positive (1/2) |  | 24(82.8) | 102(76.1) | 31(88.6) | 95(74.2) | 30(85.7) | 90(76.9) | 75(82.4) | 45(73.8) | -- | -- |
| Unknown |  | 3(10.3) | 16(11.9) | 2(5.7) | 17(13.3) | 5(14.3) | 8(6.8) | 7(7.7) | 6(9.8) | -- | -- |

Abbreviations: BMI: body mass index, BBD: benign breast disease

**Supplementary Table 2.** Participant and tumor characteristics by case and tumor expression status among postmenopausal women

|  |  | **Tumor Characteristics** | | | | | | | | | |
| --- | --- | --- | --- | --- | --- | --- | --- | --- | --- | --- | --- |
| **Participant Characteristics** | **Controls** | **Prolactin receptor Nuclear** | | **Prolactin receptor Cytoplasmic** | | **pSTAT5**  **Nuclear** | | **pSTAT5**  **Cytoplasmic** | | **pJAK2** | |
|  | **N=1689** | **Positive (2/3)**  **N=139** | **Negative (0/1)**  **N=406** | **Positive (2/3)**  **N=129** | **Negative (0/1)**  **N=416** | **Positive (2/3)**  **N=125** | **Negative (0/1)**  **N=394** | **Positive (1/2)**  **N=306** | **Negative (0)**  **N=213** | **Positive (1/2)**  **N=409** | **Negative (0)**  **N=95** |
|  | **Mean (SD)** | | | | | | | | | | |
| Age at blood collection | 62.7(6.3) | 63.1(6.1) | 62.6(6.7) | 63.5(7.8) | 62.5(6.1) | 62.1(6.9) | 62.8(6.5) | 62.9(6.7) | 62.3(6.4) | 62.7(6.6) | 64.3(6.9) |
| Age at menarche | 12.6(1.4) | 12.6(1.4) | 12.4(1.3) | 12.4(1.3) | 12.5(1.4) | 12.6(1.2) | 12.5(1.4) | 12.5(1.3) | 12.5(1.4) | 12.6(1.4) | 12.3(1.2) |
| BMI at blood collection | 25.8(4.5) | 26(4.8) | 26(4.6) | 27.3(5.0) | 25.6(4.5) | 25.6(4.8) | 26.1(4.7) | 25.9(4.5) | 26.1(5.0) | 25.9(4.8) | 25.8(4.2) |
| Prolactin (ng/mL) | 11.4(7.7) | 13.2(10.8) | 11.6(6.4) | 12.2(8.0) | 11.9(7.7) | 12.8(8.5) | 11.9(7.6) | 12.2(7.4) | 11.9(8.4) | 12(7.7) | 12.4(7.7) |
|  | **N (%)** | | | | | | | | | | |
| Fasting at blood collection | 1205(71.3) | 100(71.9) | 290(71.4) | 90(69.8) | 300(72.1) | 86(68.8) | 287(72.8) | 222(72.5) | 151(70.9) | 283(69.2) | 72(75.8) |
| Family history of breast cancer | 224(13.3) | 28(20.1) | 68(16.7) | 21(16.3) | 75(18.0) | 15(12) | 72(18.3) | 45(14.7) | 42(19.7) | 66(16.1) | 19(20.0) |
| History of BBD |  |  |  |  |  |  |  |  |  |  |  |
| Biopsy-confirmed | 276(16.3) | 28(20.1) | 84(20.7) | 33(25.6) | 79(19.0) | 25(20.0) | 85(21.6) | 66(21.6) | 44(20.7) | 89(21.8) | 27(28.4) |
| Unconfirmed or confirmation unknown | 485(28.7) | 41(29.5) | 138(34.0) | 37(28.7) | 142(34.1) | 44(35.2) | 127(32.2) | 94(30.7) | 77(36.2) | 137(33.5) | 26(27.4) |
| HT use |  |  |  |  |  |  |  |  |  |  |  |
| No HT use | 968(57.3) | 52(37.4) | 166(40.9) | 46(35.7) | 172(41.3) | 41(32.8) | 166(42.1) | 116(37.9) | 91(42.7) | 162(39.6) | 37(38.9) |
| HT use | 721(42.7) | 87(62.6) | 240(59.1) | 83(64.3) | 244(58.7) | 84(67.2) | 228(57.9) | 190(62.1) | 122(57.3) | 247(60.4) | 58(61.1) |
| **Tumor characteristics** |  |  |  |  |  |  |  |  |  |  |  |
| Invasiveness |  |  |  |  |  |  |  |  |  |  |  |
| In situ |  | 30(21.6) | 43(10.6) | 18(14.0) | 55(13.2) | 20(16.0) | 55(14.0) | 53(17.3) | 22(10.3) | 59(14.4) | 11(11.6) |
| Invasive |  | 105(75.5) | 355(87.4) | 108(83.7) | 352(84.6) | 102(81.6) | 330(83.8) | 244(79.7) | 188(88.3) | 341(83.4) | 83(87.4) |
| Unknown |  | 4(2.9) | 8(2.0) | 3(2.3) | 9(2.2) | 3(2.4) | 9(2.3) | 9(2.9) | 3(1.4) | 9(2.2) | 1(1.1) |
| Differentiation |  |  |  |  |  |  |  |  |  |  |  |
| Well or moderately differentiated |  | 62(44.6) | 175(43.1) | 45(34.9) | 192(46.2) | 60(48.0) | 161(40.9) | 132(43.1) | 89(41.8) | 170(41.6) | 48(50.5) |
| Poorly differentiated |  | 16(11.5) | 108(26.6) | 49(38.0) | 75(18.0) | 16(12.8) | 100(25.4) | 61(19.9) | 55(25.8) | 89(21.8) | 27(28.4) |
| Unknown |  | 61(43.9) | 123(30.3) | 35(27.1) | 149(35.8) | 49(39.2) | 133(33.8) | 113(36.9) | 69(32.4) | 150(36.7) | 20(21.1) |
| Lymph node status |  |  |  |  |  |  |  |  |  |  |  |
| No lymph nodes |  | 84(60.4) | 273(67.2) | 81(62.8) | 276(66.3) | 80(64.0) | 256(65.0) | 185(60.5) | 151(70.9) | 265(64.8) | 60(63.2) |
| At least 1 lymph node involved |  | 21(15.1) | 82(20.2) | 27(20.9) | 76(18.3) | 22(17.6) | 74(18.8) | 59(19.3) | 37(17.4) | 76(18.6) | 23(24.2) |
| Unknown |  | 34(24.5) | 51(12.6) | 21(16.3) | 64(15.4) | 23(18.4) | 64(16.2) | 62(20.3) | 25(11.7) | 68(16.6) | 12(12.6) |
| Tumor size |  |  |  |  |  |  |  |  |  |  |  |
| <2 cm |  | 81(58.3) | 249(61.3) | 73(56.6) | 257(61.8) | 71(56.8) | 240(60.9) | 178(58.2) | 133(62.4) | 251(61.4) | 55(57.9) |
| ≥2cm |  | 23(16.5) | 98(24.1) | 34(26.4) | 87(20.9) | 26(20.8) | 86(21.8) | 60(19.6) | 52(24.4) | 81(19.8) | 28(29.5) |
| Unknown |  | 35(25.2) | 59(14.5) | 22(17.1) | 72(17.3) | 28(22.4) | 68(17.3) | 68(22.2) | 28(13.1) | 77(18.8) | 12(12.6) |
| Ductal vs lobular |  |  |  |  |  |  |  |  |  |  |  |
| Ductal |  | 81(58.3) | 294(72.4) | 103(79.8) | 272(65.4) | 86(68.8) | 265(67.3) | 204(66.7) | 147(69.0) | 282(68.9) | 67(70.5) |
| Lobular |  | 17(12.2) | 37(9.1) | -- | 54(13.0) | 13(10.4) | 36(9.1) | 21(6.9) | 28(13.1) | 34(8.3) | 8(8.4) |
| Unknown |  | 41(29.5) | 75(18.5) | 26(20.2) | 90(21.6) | 26(20.8) | 93(23.6) | 81(26.5) | 38(17.8) | 93(22.7) | 20(21.1) |
| Estrogen receptor |  |  |  |  |  |  |  |  |  |  |  |
| Negative |  | 11(7.9) | 89(21.9) | 54(41.9) | 46(11.1) | 18(14.4) | 79(20.1) | 56(18.3) | 41(19.2) | 77(18.8) | 17(17.9) |
| Positive |  | 126(90.6) | 312(76.8) | 74(57.4) | 364(87.5) | 105(84.0) | 308(78.2) | 245(80.1) | 168(78.9) | 325(79.5) | 78(82.1) |
| Unknown |  | 2(1.4) | 5(1.2) | 1(0.8) | 6(1.4) | 2(1.6) | 7(1.8) | 5(1.6) | 4(1.9) | 7(1.7) | -- |
| Progesterone receptor |  |  |  |  |  |  |  |  |  |  |  |
| Negative |  | 27(19.4) | 115(28.3) | 57(44.2) | 85(20.4) | 27(21.6) | 108(27.4) | 80(26.1) | 55(25.8) | 104(25.4) | 24(25.3) |
| Positive |  | 111(79.9) | 288(70.9) | 71(55) | 328(78.8) | 96(76.8) | 283(71.8) | 223(72.9) | 156(73.2) | 301(73.6) | 70(73.7) |
| Unknown |  | 1(0.7) | 3(0.7) | 1(0.8) | 3(0.7) | 2(1.6) | 3(0.8) | 3(1.0) | 2(0.9) | 4(1.0) | 1(1.1) |
| HER-2 |  |  |  |  |  |  |  |  |  |  |  |
| Negative |  | 120(86.3) | 290(71.4) | 86(66.7) | 324(77.9) | 104(83.2) | 289(73.4) | 207(67.6) | 186(87.3) | 313(76.5) | 64(67.4) |
| Positive |  | 17(12.2) | 106(26.1) | 42(32.6) | 81(19.5) | 17(13.6) | 96(24.4) | 94(30.7) | 19(8.9) | 91(22.2) | 28(29.5) |
| Unknown |  | 2(1.4) | 10(2.5) | 1(0.8) | 11(2.6) | 4(3.2) | 9(2.3) | 5(1.6) | 8(3.8) | 5(1.2) | 3(3.2) |
| Prolactin Receptor Nuclear |  |  |  |  |  |  |  |  |  |  |  |
| Negative (0/1) |  | -- | -- | 108(83.7) | 298(71.6) | 72(57.6) | 296(75.1) | 194(63.4) | 174(81.7) | 285(69.7) | 72(75.8) |
| Positive (2/3) |  | -- | -- | 21(16.3) | 118(28.4) | 48(38.4) | 85(21.6) | 102(33.3) | 31(14.6) | 103(25.2) | 20(21.1) |
| Unknown |  | -- | -- | -- | -- | 5(4.0) | 13(3.3) | 10(3.3) | 8(3.8) | 21(5.1) | 3(3.2) |
| Prolactin Receptor Cytoplasmic |  |  |  |  |  |  |  |  |  |  |  |
| Negative (0/1) |  | 118(84.9) | 298(73.4) | -- | -- | 90(72) | 287(72.8) | 205(67.0) | 172(80.8) | 283(69.2) | 69(72.6) |
| Positive (2/3) |  | 21(15.1) | 108(26.6) | -- | -- | 30(24) | 94(23.9) | 91(29.7) | 33(15.5) | 105(25.7) | 23(24.2) |
| Unknown |  | -- | -- | -- | -- | 5(4.0) | 13(3.3) | 10(3.3) | 8(3.8) | 21(5.1) | 3(3.2) |
| pSTAT5 Nuclear |  |  |  |  |  |  |  |  |  |  |  |
| Negative (0/1) |  | 85(61.2) | 296(72.9) | 94(72.9) | 287(69.0) | -- | -- | 205(67.0) | 189(88.7) | 269(65.8) | 79(83.2) |
| Positive (2/3) |  | 48(34.5) | 72(17.7) | 30(23.3) | 90(21.6) | -- | -- | 101(33.0) | 24(11.3) | 107(26.2) | 9(9.5) |
| Unknown |  | 6(4.3) | 38(9.4) | 5(3.9) | 39(9.4) | -- | -- | -- | -- | 33(8.1) | 7(7.4) |
| pSTAT5 Cytoplasmic |  |  |  |  |  |  |  |  |  |  |  |
| Negative (0) |  | 31(22.3) | 174(42.9) | 33(25.6) | 172(41.3) | 24(19.2) | 189(48.0) | -- | -- | 133(32.5) | 48(50.5) |
| Positive (1/2) |  | 102(73.4) | 194(47.8) | 91(70.5) | 205(49.3) | 101(80.8) | 205(52.0) | -- | -- | 243(59.4) | 40(42.1) |
| Unknown |  | 6(4.3) | 38(9.4) | 5(3.9) | 39(9.4) | -- | -- | -- | -- | 33(8.1) | 7(7.4) |
| pJAK2 |  |  |  |  |  |  |  |  |  |  |  |
| Negative (0) |  | 20(14.4) | 72(17.7) | 23(17.8) | 69(16.6) | 9(7.2) | 79(20.1) | 40(13.1) | 48(22.5) | -- | -- |
| Positive (1/2) |  | 103(74.1) | 285(70.2) | 105(81.4) | 283(68.0) | 107(85.6) | 269(68.3) | 243(79.4) | 133(62.4) | -- | -- |
| Unknown |  | 16(11.5) | 49(12.1) | 1(0.8) | 64(15.4) | 9(7.2) | 46(11.7) | 23(7.5) | 32(15.0) | -- | -- |

Abbreviations: BMI: body mass index, BBD: benign breast disease, HT: hormone therapy

**Supplemental Table 3.** Odds ratios (ORs) and 95% confidence intervals (CIs) for breast cancer comparing plasma prolactin >11 versus ≤11 ng/ml by joint tumor expression of prolactin-related markers among postmenopausal women

|  | **Prolactin receptor cytoplasmic** ^a^ | | | **pSTAT5 Nuclear** ^a^ | | | **pSTAT5 Cytoplasmic** ^b^ | | | **pJAK2** ^b^ | | |
| --- | --- | --- | --- | --- | --- | --- | --- | --- | --- | --- | --- | --- |
|  | Negative | Positive | p-het | Negative | Positive | p-het | Negative | Positive | p-het | Negative | Positive | p-het |
| **Prolactin receptor nuclear** ^a^ | |  | **0.05** |  |  | 0.49 |  |  | 0.70 |  |  | 0.48 |
| Case Ns | 298 | 108 |  | 296 | 72 |  | 174 | 194 |  | 72 | 285 |  |
| Negative | 1.4(1.1,1.8) | 1.2(0.8,1.8) |  | 1.2(1.0,1.6) | 1.8(1.1,2.9) |  | 1.5(1.1,2.0) | 1.2(0.9,1.6) |  | 1.3(0.8,2.1) | 1.3(1.0,1.7) |  |
| Case Ns | 118 | 21 |  | 85 | 48 |  | 31 | 102 |  | 20 | 103 |  |
| Positive | 1.2(0.8,1.7) | 5.9(1.9,18.3)* |  | 1.5(0.9,2.3) | 1.7(0.9,3.0) |  | 1.4(0.7,2.9) | 1.6(1.0,2.4) |  | 2.6(1.0,6.7) | 1.6(1.0,2.3) |  |
| **Prolactin receptor cytoplasmic** ^a^ | |  |  |  |  | 0.52 |  |  | 0.88 |  |  | 0.66 |
| Case Ns |  |  |  | 287 | 90 |  | 172 | 205 |  | 69 | 283 |  |
| Negative |  |  |  | 1.2(1.0,1.6) | 1.7(1.1,2.7) |  | 1.5(1.0,2.0) | 1.3(0.9,1.7) |  | 1.7(1.0,2.7) | 1.3(1.0,1.7) |  |
| Case Ns |  |  |  | 94 | 30 |  | 33 | 91 |  | 23 | 105 |  |
| Positive |  |  |  | 1.4(0.9,2.2) | 1.7(0.8,3.5) |  | 1.5(0.7,3.1) | 1.5(1.0,2.3) |  | 1.1(0.5,2.5) | 1.6(1.0,2.3) |  |
| **pSTAT5 Nuclear** ^a^ |  |  |  |  |  |  |  |  | 0.57 |  |  | 0.41 |
| Case Ns |  |  |  |  |  |  | 189 | 205 |  | 79 | 269 |  |
| Negative |  |  |  |  |  |  | 1.4(1.0,1.9) | 1.2(0.9,1.6) |  | 1.7(1.1,2.8) | 1.2(1.0,1.6) |  |
| Case Ns |  |  |  |  |  |  | 24 | 101 |  | 9 | 107 |  |
| Positive |  |  |  |  |  |  | 1.9(0.8,4.4) | 1.6(1.1,2.5) |  | 1.4(0.4,5.7) | 1.7(1.2,2.6) |  |
| **pSTAT5 Cytoplasmic** ^b^ |  |  |  |  |  |  |  |  |  |  |  | **0.07** |
| Case Ns |  |  |  |  |  |  |  |  |  | 48 | 133 |  |
| Negative |  |  |  |  |  |  |  |  |  | 2.8(1.5,5.1) | 1.2(0.8,1.7) |  |
| Case Ns |  |  |  |  |  |  |  |  |  | 40 | 243 |  |
| Positive |  |  |  |  |  |  |  |  |  | 1.0(0.5,1.9) | 1.5(1.1,2.0) |  |

Adjusted for age (continuous), age at menarche (continuous), BMI (continuous), benign breast disease (never, confirmed, unconfirmed), HT status (no, yes), and blood draw characteristics (fasting status and month of blood collection).

Abbreviations: p-het: p for heterogeneity

*pairwise comparisons to other 3 subtypes p≤0.01

^a^ A case was considered positive if one or more cores were scored 2 or 3

^b^ Cytoplasmic pSTAT5 and pJAK2 was scored positive if any core was scored 1 or higher.

**Supplemental Table 4.** Odds ratios (ORs) and 95% confidence intervals (CIs) for breast cancer comparing plasma prolactin ≥11 versus <11 ng/ml by tumor expression of prolactin-related markers premenopausal women with invasive tumors

|  | | **Prolactin levels (ng/ml)** | |  |
| --- | --- | --- | --- | --- |
| **Tumor status** | **N (Cases)** | ≤**11** | **>11** | **p-heterogeneity** |
| **All breast cancer** | 140 | 1(Ref) | **1.20 (0.82, 1.77)** |  |
| **Prolactin receptor** |  |  |  |  |
| Nuclear ^a^ |  |  |  |  |
| Negative | 110 | 1 (Ref) | 1.19 (0.78, 1.82) | 0.84 |
| Positive | 25 | 1 (Ref) | 1.31 (0.56, 3.06) |  |
| Cytoplasmic ^a^ |  |  |  |  |
| Negative | 107 | 1 (Ref) | 1.41 (0.91, 2.20) | 0.12 |
| Positive | 28 | 1 (Ref) | 0.71 (0.33, 1.55) |  |
| **pSTAT5** |  |  |  |  |
| Nuclear ^a^ |  |  |  |  |
| Negative | 97 | 1 (Ref) | 1.00 (0.64, 1.56) | 0.14 |
| Positive | 29 | 1 (Ref) | 2.09 (0.86, 5.05) |  |
| Cytoplasmic ^b^ |  |  |  |  |
| Negative | 51 | 1 (Ref) | 0.68 (0.38, 1.21) | **0.01** |
| Positive | 75 | 1 (Ref) | **1.77 (1.03, 3.03)** |  |
| **pJAK2** ^b^ |  |  |  |  |
| Negative | 18 | 1 (Ref) | 1.52 (0.55, 4.22) | 0.69 |
| Positive | 104 | 1 (Ref) | 1.22 (0.79, 1.89) |  |

Adjusted for age (continuous), age at menarche (continuous), BMI (continuous), benign breast disease (never, confirmed, unconfirmed), HT status (no, yes), and blood draw characteristics (fasting status and month of blood collection)

^a^ A case was considered positive if one or more cores were scored 2 or 3

^b^ Cytoplasmic pSTAT5 and pJAK2 was scored positive if any core was scored 1 or higher.

**Supplemental Table 5.** Odds ratios (ORs) and 95% confidence intervals (CIs) for breast cancer comparing plasma prolactin ≥11 versus <11 ng/ml by tumor expression of prolactin-related markers postmenopausal women with invasive tumors

|  | | **Prolactin levels (ng/ml)** | |  |
| --- | --- | --- | --- | --- |
| **Tumor status** | **N (Cases)** | ≤**11** | **>11** | **p-heterogeneity** |
| **All breast cancer** | 495 | 1(Ref) | **1.39 (1.13, 1.72)** |  |
| **Prolactin receptor** |  |  |  |  |
| Nuclear ^a^ |  |  |  |  |
| Negative | 363 | 1 (Ref) | **1.36 (1.08, 1.73)** | 0.92 |
| Positive | 109 | 1 (Ref) | 1.40 (0.94, 2.08) |  |
| Cytoplasmic ^a^ |  |  |  |  |
| Negative | 361 | 1 (Ref) | **1.37 (1.08, 1.74)** | >0.99 |
| Positive | 111 | 1 (Ref) | 1.37 (0.92, 2.05) |  |
| **pSTAT5** |  |  |  |  |
| Nuclear ^a^ |  |  |  |  |
| Negative | 339 | 1 (Ref) | **1.35 (1.06, 1.72)** | 0.49 |
| Positive | 105 | 1 (Ref) | **1.58 (1.05, 2.37)** |  |
| Cytoplasmic ^b^ |  |  |  |  |
| Negative | 191 | 1 (Ref) | **1.56 (1.15, 2.13)** | 0.32 |
| Positive | 253 | 1 (Ref) | 1.29 (0.98, 1.69) |  |
| **pJAK2** ^b^ |  |  |  |  |
| Negative | 84 | 1 (Ref) | 1.56 (0.99, 2.45) | 0.60 |
| Positive | 350 | 1 (Ref) | **1.37 (1.07, 1.74)** |  |

Adjusted for age (continuous), age at menarche (continuous), BMI (continuous), benign breast disease (never, confirmed, unconfirmed), HT status (no, yes), and blood draw characteristics (fasting status and month of blood collection)

^a^ A case was considered positive if one or more cores were scored 2 or 3

^b^ Cytoplasmic pSTAT5 and pJAK2 was scored positive if any core was scored 1 or higher.

**Supplemental Table 6.** Odds ratios (ORs) and 95% confidence intervals (CIs) for breast cancer comparing plasma prolactin ≥11 versus <11 ng/ml by tumor expression of prolactin-related markers among postmenopausal **and** ER+/PR+ tumors

|  | | **Prolactin levels (ng/ml)** | |  |
| --- | --- | --- | --- | --- |
| **Tumor status** | **N (Cases)** | ≤**11** | **>11** | **p-heterogeneity** |
| **All breast cancer** | 478 | 1(Ref) | **1.36 (1.10, 1.68)** |  |
| **Prolactin receptor** |  |  |  |  |
| Nuclear ^a^ |  |  |  |  |
| Negative | 323 | 1 (Ref) | **1.33 (1.04, 1.70)** | 0.82 |
| Positive | 129 | 1 (Ref) | 1.39 (0.96, 2.02) |  |
| Cytoplasmic ^a^ |  |  |  |  |
| Negative | 373 | 1 (Ref) | **1.33 (1.06, 1.68)** | 0.87 |
| Positive | 79 | 1 (Ref) | 1.39 (0.86, 2.24) |  |
| **pSTAT5** |  |  |  |  |
| Nuclear ^a^ |  |  |  |  |
| Negative | 320 | 1 (Ref) | **1.30 (1.01, 1.67)** | 0.43 |
| Positive | 107 | 1 (Ref) | **1.56 (1.04, 2.34)** |  |
| Cytoplasmic ^b^ |  |  |  |  |
| Negative | 174 | 1 (Ref) | **1.52 (1.10, 2.10)** | 0.34 |
| Positive | 253 | 1 (Ref) | 1.26 (0.96, 1.66) |  |
| **pJAK2** ^b^ |  |  |  |  |
| Negative | 80 | 1 (Ref) | 1.56 (0.98, 2.48) | 0.68 |
| Positive | 336 | 1 (Ref) | **1.41 (1.10, 1.80)** |  |

Adjusted for age (continuous), age at menarche (continuous), BMI (continuous), benign breast disease (never, confirmed, unconfirmed), HT status (no, yes), and blood draw characteristics (fasting status and month of blood collection)

^a^ A case was considered positive if one or more cores were scored 2 or 3

^b^ Cytoplasmic pSTAT5 and pJAK2 was scored positive if any core was scored 1 or higher.

**Supplemental Table 7.** Tumor characteristics among **premenopausal** cases by tumor expression of prolactin-related markers

|  | Prolactin Receptor Nuclear | | Prolactin Receptor Cytoplasmic | | pSTAT5 Nuclear | | pSTAT5 Cytoplasmic | | pJAK2 | |
| --- | --- | --- | --- | --- | --- | --- | --- | --- | --- | --- |
|  | Negative (n=134) | Positive (n=29) | Negative (n=128) | Positive (n=35) | Negative (n=117) | Positive (n=35) | Negative (n=61) | Positive (n=91) | Negative (n=20) | Positive (n=127) |
| Ductal/Lobular ^a^ |  |  |  |  |  |  |  |  |  |  |
| Ductal | 110 (82.1) | 21 (72.4) | 99 (77.3) | 32 (91.4) | 92 (78.6) | 31 (91.2) | 48 (80.0) | 75 (82.4) | 16 (80.0) | 102 (80.3) |
| Lobular | 19 (14.2) | 8 (27.6) | 25 (19.5) | 2 (5.7) | 20 (17.1) | 3 (8.8) | 8 (13.3) | 15 (16.5) | 3 (15.0) | 22 (17.3) |
| Both | 5 (3.7) | 0 (0) | 4 (3.1) | 1 (2.9) | 5 (4.3) | 0 (0) | 4 (6.7) | 1 (1.1) | 1 (5.0) | 3 (2.4) |
| Lymph Nodes |  |  |  |  |  |  |  |  |  |  |
| 0 Nodes Involved | 100 (74.6) | 20 (69.0) | 94 (73.4) | 26 (74.3) | 80 (68.4) | 30 (85.7) | 43 (70.5) | 67 (73.6) | 11 (55.0) | 98 (77.2) |
| 1-3 Nodes Involved | 17 (12.7) | 8 (27.6) | 20 (15.6) | 5 (14.3) | 22 (18.8) | 3 (8.6) | 9 (14.8) | 16 (17.6) | 5 (25.0) | 18 (14.2) |
| 4+ Nodes Involved / Metastasis at Diagnosis | 17 (12.7) | 1 (3.5) | 14 (10.9) | 4 (11.4) | 15 (12.8) | 2 (5.7) | 9 (14.8) | 8 (8.8) | 4 (20.0) | 11 (8.7) |
| Tumor Grade ^b^ |  |  |  |  |  |  |  |  |  |  |
| Well-Differentiated | 11 (13.3) | 2 (12.5) | 12 (16.2) | 1 (4.0) | 9 (12.7) | 4 (17.4) | 3 (7.5) | 10 (18.5) | 1 (6.7) | 12 (16.0) |
| Moderately Differentiated | 37 (44.6) | 11 (68.8) | 39 (52.7) | 9 (36.0) | 33 (46.5) | 11 (47.8) | 19 (47.5) | 25 (46.3) | 5 (33.3) | 36 (48.0) |
| Poorly Differentiated | 35 (42.2) | 3 (18.8) | 23 (31.1) | 15 (60.0) | 29 (40.9) | 8 (34.8) | 18 (45.0) | 19 (35.2) | 9 (60.0) | 27 (36.0) |
| Tumor Size ^c^ |  |  |  |  |  |  |  |  |  |  |
| ≤2.0 cm | 85 (74.6) | 18 (78.3) | 76 (71.7) | 27 (87.1) | 74 (74.8) | 25 (83.3) | 43 (84.3) | 56 (71.8) | 11 (64.7) | 84 (79.3) |
| 2.1-4.0 cm | 15 (13.2) | 4 (17.4) | 15 (14.2) | 4 (12.9) | 13 (13.1) | 4 (13.3) | 4 (7.8) | 13 (16.7) | 2 (11.8) | 15 (14.2) |
| >4.0 cm | 14 (12.3) | 1 (4.4) | 15 (14.2) | 0 (0) | 12 (12.1) | 1 (3.3) | 4 (7.8) | 9 (11.5) | 4 (23.5) | 7 (6.6) |

^a^ Unknown for 1 cases

^b^ Unknown for 65 cases

^c^ Unknown for 26 cases

**Supplemental Table 8.** Tumor characteristics among **postmenopausal** cases by tumor expression of prolactin-related markers

|  | Prolactin Receptor Nuclear | | Prolactin Receptor Cytoplasmic | | pSTAT5 Nuclear | | pSTAT5 Cytoplasmic | | pJAK2 | |
| --- | --- | --- | --- | --- | --- | --- | --- | --- | --- | --- |
|  | Negative (n=406) | Positive (n=138) | Negative (n=416) | Positive (n=129) | Negative (n=394) | Positive (n=125) | Negative (n=213) | Positive (n=306) | Negative (n=95) | Positive (n=409) |
| Ductal/Lobular ^a^ |  |  |  |  |  |  |  |  |  |  |
| Ductal | 338 (84.1) | 110 (79.7) | 330 (79.5) | 118 (94.4) | 320 (82.3) | 105 (84.0) | 169 (80.5) | 256 (84.2) | 77 (81.9) | 340 (84.0) |
| Lobular | 40 (10.0) | 21 (15.2) | 58 (14.0) | 3 (2.4) | 40 (10.3) | 17 (13.6) | 29 (13.8) | 28 (9.2) | 8 (8.5) | 41 (10.1) |
| Both | 24 (6.0) | 7 (5.1) | 27 (6.5) | 4 (3.2) | 29 (7.5) | 3 (2.4) | 12 (5.7) | 20 (6.6) | 9 (9.6) | 24 (5.9) |
| Lymph Nodes |  |  |  |  |  |  |  |  |  |  |
| 0 Nodes Involved | 322 (79.3) | 117 (84.2) | 339 (81.5) | 100 (77.5) | 318 (80.7) | 102 (81.6) | 176 (82.6) | 244 (79.7) | 71 (74.7) | 331 (80.9) |
| 1-3 Nodes Involved | 62 (15.3) | 16 (11.5) | 52 (12.5) | 26 (20.2) | 53 (13.5) | 18 (14.4) | 25 (11.7) | 46 (15.0) | 18 (19.0) | 56 (13.7) |
| 4+ Nodes Involved / Metastasis at Diagnosis | 22 (5.4) | 6 (4.3) | 25 (6.0) | 3 (2.3) | 23 (5.8) | 5 (4.0) | 12 (5.6) | 16 (5.2) | 6 (6.3) | 22 (5.4) |
| Tumor Grade ^b^ |  |  |  |  |  |  |  |  |  |  |
| Well-Differentiated | 67 (23.3) | 28 (34.2) | 82 (29.9) | 13 (13.5) | 59 (22.2) | 24 (30.0) | 31 (21.2) | 52 (26.0) | 22 (29.3) | 69 (25.8) |
| Moderately Differentiated | 113 (39.2) | 38 (46.3) | 117 (42.7) | 34 (35.4) | 108 (40.6) | 39 (48.8) | 61 (41.8) | 86 (43.0) | 27 (36.0) | 108 (40.5) |
| Poorly Differentiated | 108 (37.5) | 16 (19.5) | 75 (27.4) | 49 (51.0) | 99 (37.2) | 17 (21.3) | 54 (37.0) | 62 (31.0) | 26 (34.7) | 90 (33.7) |
| Tumor Size ^c^ |  |  |  |  |  |  |  |  |  |  |
| ≤2.0 cm | 267 (72.8) | 89 (78.8) | 282 (76.2) | 74 (67.3) | 258 (74.4) | 77 (74.8) | 141 (72.7) | 194 (75.8) | 56 (66.7) | 271 (76.3) |
| 2.1-4.0 cm | 88 (24.0) | 21 (18.6) | 76 (20.5) | 33 (30.0) | 78 (22.5) | 23 (22.3) | 49 (25.3) | 52 (20.3) | 28 (33.3) | 70 (19.7) |
| >4.0 cm | 12 (3.3) | 3 (2.7) | 12 (3.2) | 3 (2.7) | 11 (3.2) | 3 (2.9) | 4 (2.1) | 10 (3.9) | 0 (0) | 14 (3.9) |

^a^ Unknown for 5 cases

^b^ Unknown for 194 cases

^c^ Unknown for 74 cases

**Supplemental Figure 1.** Odds ratios (ORs) and 95% confidence intervals (CIs) for breast cancer comparing plasma prolactin ≥11 versus <11 ng/ml by tumor expression of prolactin-related markers a) PRLR, b) pSTAT5, and c) pJAK2
